# Chicago Doctors

**Published:** 1881-10

**Authors:** 


CHICAGO DOCTORS.—Dr. A. H. Foster has furnished us with the following interesting statistics concerning the number of physicians and surgeons in this city, recognized as regular and in good standing by registration in the *Chicago Medical Register* for 1881. The whole number of names in the said register is 452. Of these, 234 are graduates from the regular medical colleges in Chicago; namely, eighteen from the Woman's Hospital Medical College, eighty-six from the Chicago Medical College, and 130 from the Rush Medical College. This would give one regular physician for every 1,112 inhabitants. Lest there should be some benevolent individuals who might fear this ratio indicated an inadequate supply of doctors in our city, we will state that the *Chicago City Directory* (Lakeside) for 1881, has the names of 892 persons claiming the title of "Doctor." This would indicate a doctor of some sort for every 563 inhabitants.

